# Ultrasonography of the Kidney: A Pictorial Review

**DOI:** 10.3390/diagnostics6010002

**Published:** 2015-12-23

**Authors:** Kristoffer Lindskov Hansen, Michael Bachmann Nielsen, Caroline Ewertsen

**Affiliations:** Department of Radiology, Copenhagen University Hospital, Blegdamsvej 9, Copenhagen 2100-DK, Denmark; mbn@dadlnet.dk (M.B.N.); caroline.ewertsen@dadlnet.dk (C.E.)

**Keywords:** renal ultrasound, pictorial review, practical guide

## Abstract

Ultrasonography of the kidneys is essential in the diagnosis and management of kidney-related diseases. The kidneys are easily examined, and most pathological changes in the kidneys are distinguishable with ultrasound. In this pictorial review, the most common findings in renal ultrasound are highlighted.

## 1. Introduction

Renal ultrasound (US) is a common examination, which has been performed for decades [1]. Using B-mode imaging, assessment of renal anatomy is easily performed, and US is often used as image guidance for renal interventions. Furthermore, novel applications in renal US have been introduced with contrast-enhanced ultrasound (CEUS), elastography and fusion imaging. In this pictorial review, we will highlight the most common findings in renal US.

## 2. Examination Technique

The ultrasonic renal exam does not require any preparation of the patient and is usually performed with the patient in the supine position. The kidneys are examined in longitudinal and transverse scan planes with the transducer placed in the flanks. When insonation of the kidney is obscured by intestinal air, the supine scan position is combined with the lateral decubitus position with the transducer moved dorsally. Preferably, the exam is initiated in the longitudinal scan plane, parallel to the long diameter of the kidney, as the kidney is easier to distinguish [2].

In the adult patient, a curved array transducer with center frequencies of 3–6 MHz is used, while the pediatric patient should be examined with a linear array transducer with higher center frequencies. Artifacts of the lowest ribs always shadow the upper poles of the kidneys. However, the whole kidney can be examined during either normal respiration or breath hold, as the kidney will follow the diaphragm and change position accordingly [2].

## 3. Findings in the Normal Kidney

In the longitudinal scan plane, the kidney has the characteristic oval bean-shape. The right kidney is often found more caudally and is slimmer than the left kidney, which may have a so-called dromedary hump due to its proximity to the spleen [3]. The kidney is surrounded by a capsule separating the kidney from the echogenic perirenal fat, which is seen as a thin linear structure [3].

**Figure 1:**
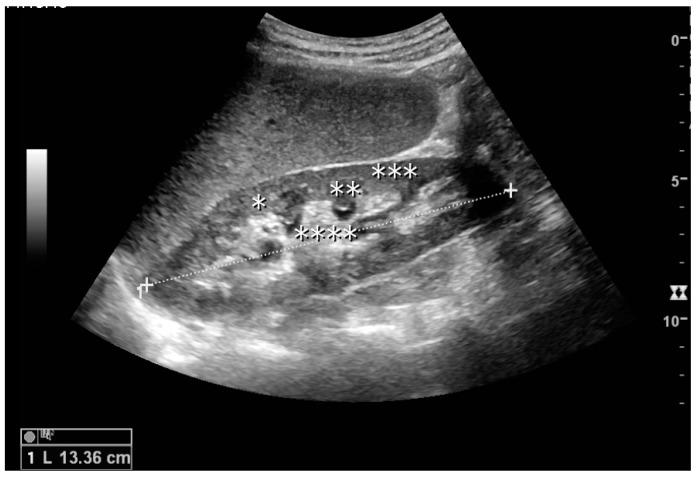
Normal adult kidney. Measurement of kidney length on the US image is illustrated by ‘+’ and a dashed line. * Column of Bertin; ** pyramid; *** cortex; **** sinus.

**Figure 2:**
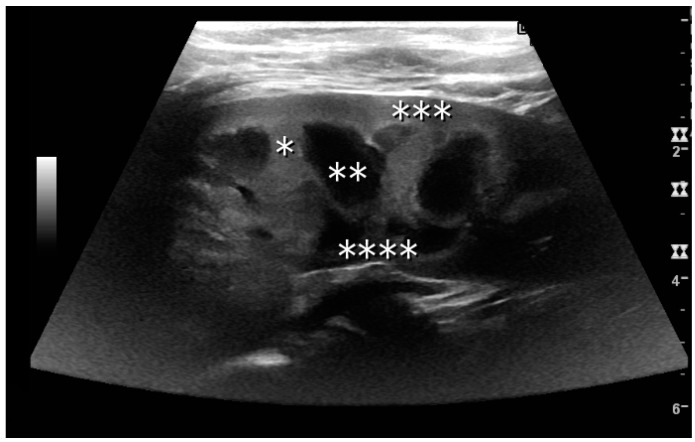
Normal pediatric kidney. * Column of Bertin; ** pyramid; *** cortex; **** sinus.

The kidney is divided into parenchyma and renal sinus. The renal sinus is hyperechoic and is composed of calyces, the renal pelvis, fat and the major intrarenal vessels. In the normal kidney, the urinary collecting system in the renal sinus is not visible, but it creates a heteroechoic appearance with the interposed fat and vessels. The parenchyma is more hypoechoic and homogenous and is divided into the outermost cortex and the innermost and slightly less echogenic medullary pyramids [3]. Between the pyramids are the cortical infoldings, called columns of Bertin (Figure 1). In the pediatric patient, it is easier to differentiate the hypoechoic medullar pyramids from the more echogenic peripheral zone of the cortex in the parenchyma rim, as well as the columns of Bertin (Figure 2) [2,4].

The length of the adult kidney is normally 10–12 cm, and the right kidney is often slightly longer than the left kidney [3,5]. The adult kidney size is variable due to the correlation with body height and age [3,5,6,7]; however, normograms for pediatric kidney size are available [7,8,9].

**Figure 3:**
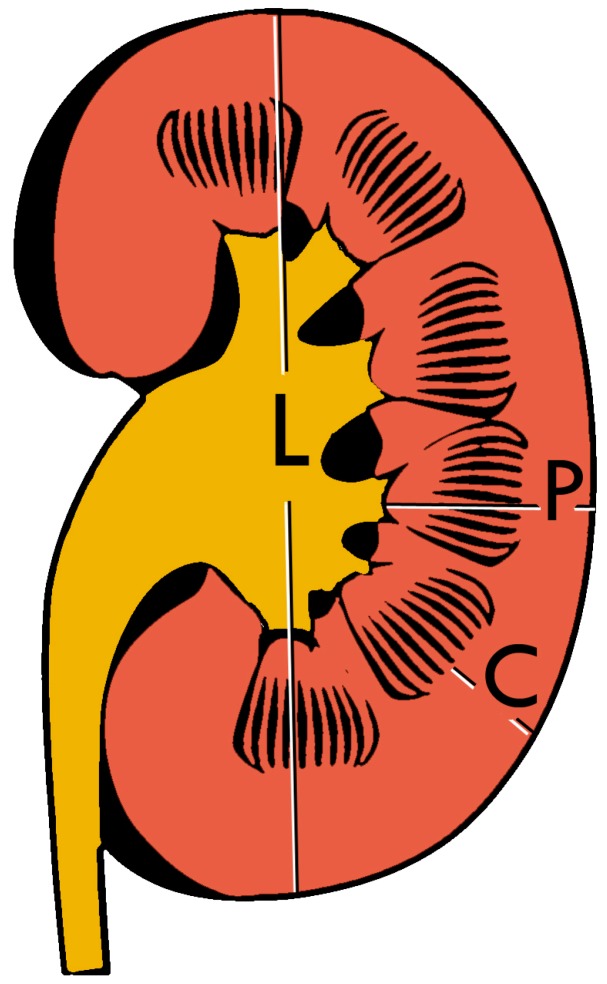
Measures of the kidney. L = length. P = parenchymal thickness. C = cortical thickness.

Cortical thickness should be estimated from the base of the pyramid and is generally 7–10 mm. If the pyramids are difficult to differentiate, the parenchymal thickness can be measured instead and should be 15–20 mm (Figure 3) [6]. The echogenicity of the cortex decreases with age and is less echogenic than or equal to the liver and spleen at the same depth in individuals older than six months [3]. In neonates and children up to six months of age, the cortex is more echogenic than the liver and spleen when compared at the same depth [10].

**Figure 4:**
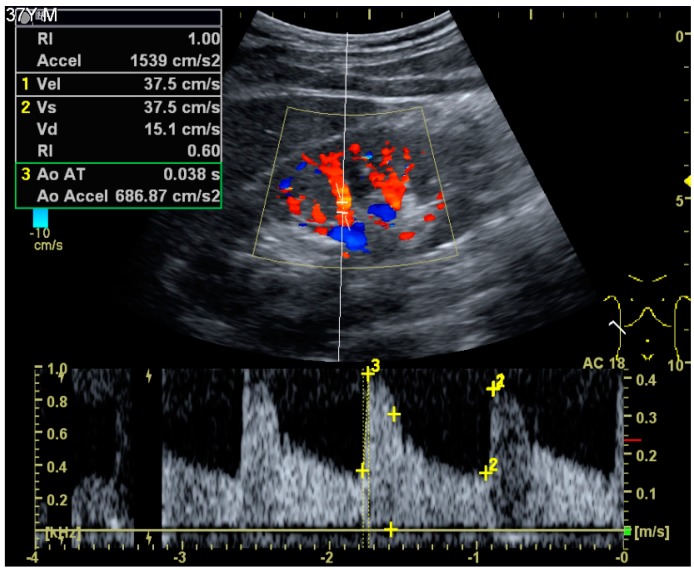
Doppler ultrasound (US) of a normal adult kidney with the estimation of the systolic velocity (Vs), the diastolic velocity (Vd), acceleration time (AoAT), systolic acceleration (Ao Accel) and resistive index (RI). Red and blue colors in the color box represent flow towards and away from the transducer, respectively. The specrogram below the B-mode image shows flow velocity (m/s) against time (s) obtained within the range gate. The small flash icons on the spectrogram represent initiation of the flow measurement.

Doppler examination of the kidney is widely used, and the vessels are easily depicted by the color Doppler technique in order to evaluate perfusion. Applying spectral Doppler to the renal artery and selected interlobular arteries, peak systolic velocities, resistive index and acceleration curves can be estimated (Figure 4) [11], e.g., peak systolic velocity of the renal artery above 180 cm/s is a predictor of renal artery stenosis of more than 60%, and the resistive index, which is a calculated from peak systolic and end systolic velocity, above 0.70 is indicative of abnormal renovascular resistance [7].

## 4. Cystic Renal Masses

**Figure 5:**
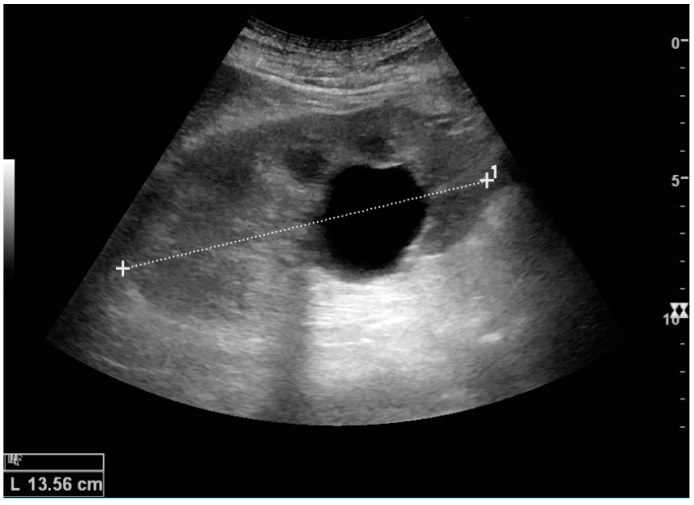
Simple cyst with posterior enhancement in an adult kidney. Measurement of kidney length on the US image is illustrated by ‘+’ and a dashed line.

Masses are seen as a distortion of the normal renal architecture. Most renal masses are simple cortical cysts with a round appearance and a smooth thin capsule encompassing anechoic fluid. The incidence increases with age, as at least 50% of people above the age of 50 have a simple cyst in one of the kidneys [12]. Cysts cause posterior enhancement as a consequence of reduced attenuation of the ultrasound within the cyst fluid (Figure 5). The simple cyst is a benign lesion, which does not require further evaluation [13].

**Figure 6:**
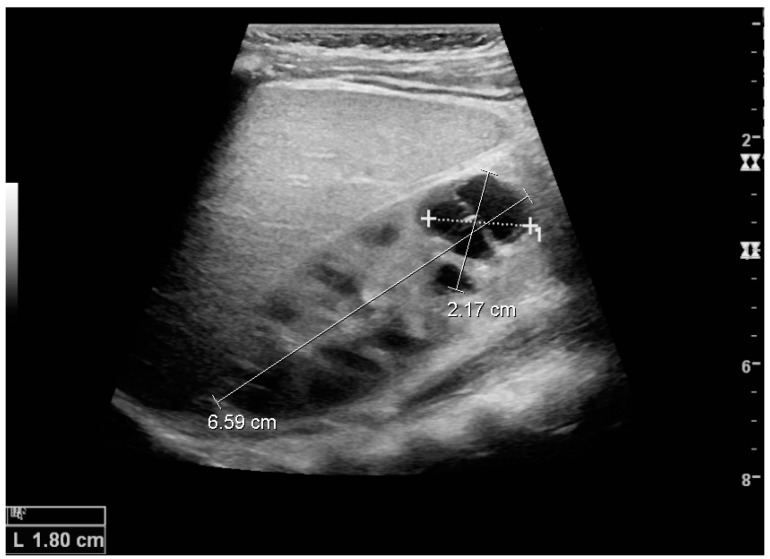
Complex cyst with thickened walls and membranes in the lower pole of an adult kidney. Measurements of kidney length and the complex cyst on the US image are illustrated by ‘+’ and dashed lines.

Complex cysts can have membranes dividing the fluid-filled center with internal echoes, calcifications or irregular thickened walls. The complex cyst can be further evaluated with Doppler US, and for Bosniak classification and follow-up of complex cysts, either contrast-enhanced ultrasound (CEUS) or contrast-enhanced computed tomography (CT) are used (Figure 6) [14,15]. The Bosniak classification is divided into four groups going from I, corresponding to a simple cyst, to IV, corresponding to a cyst with solid parts and an 85%–100% risk of malignancy [13,16].

**Figure 7:**
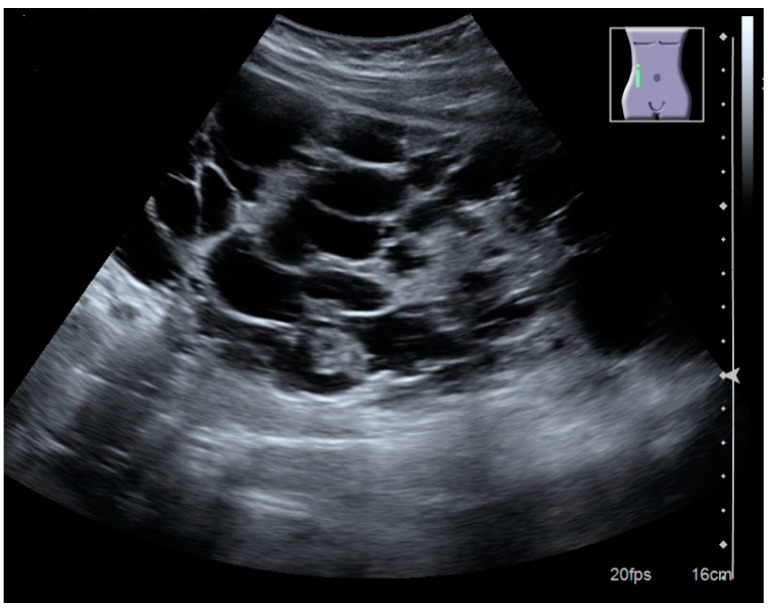
Advanced polycystic kidney disease with multiple cysts.

In polycystic kidney disease, multiple cysts of varying size in close contact with each other are seen filling virtually the entire renal region. In advanced stages of this disease, the kidneys are enlarged with a lack of corticomedullary differentiation (Figure 7) [17].

## 5. Solid Renal Masses

**Figure 8:**
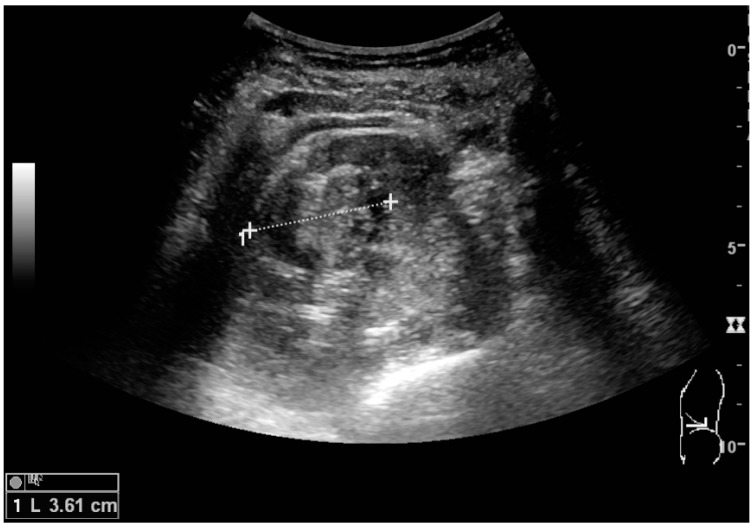
Cortical solid mass, which later was shown to be renal cell carcinoma. Measurement of the solid mass on the US image is illustrated by ‘+’ and a dashed line.

**Figure 9:**
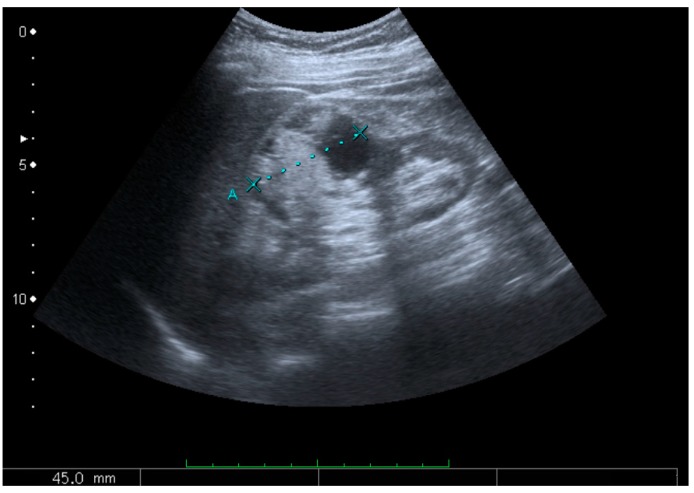
Renal cell carcinoma with both cystic and solid components located in the cortex. Measurement of tumor on the US image is illustrated by ‘+’ and a dashed line.

A solid renal mass appears in the US exam with internal echoes, without the well-defined, smooth walls seen in cysts, often with Doppler signal, and is frequently malignant or has a high malignant potential [4]. The most common malignant renal parenchymal tumor is renal cell carcinoma (RCC), which accounts for 86% of the malignancies in the kidney [2]. RCCs are typically isoechoic and peripherally located in the parenchyma, but can be both hypo- and hyper-echoic and are found centrally in medulla or sinus. The lesions can be multifocal and have cystic elements due to necrosis, calcifications and be multifocal (Figure 8 and Figure 9) [2]. RCC is associated with von Hippel–Lindau disease, and with tuberous sclerosis, and US has been recommended as a tool for assessment and follow-up of renal masses in these patients [18].

However, US is not the primary modality for the evaluation of solid tumors in the kidney, and CT is the first choice modality [19]. Nevertheless, hemorrhagic cysts can resemble RCC on CT, but they are easily distinguished with US using Doppler [19]. In RCCs, Doppler US often shows vessels with high velocities caused by neovascularization and arteriovenous shunting.

Some RCCs are hypovascular and not distinguishable with Doppler US. Therefore, renal tumors without a Doppler signal, which are not obvious simple cysts on US and CT, should be further investigated with CEUS, as CEUS is more sensitive than both Doppler US and CT for the detection of hypovascular tumors [20].

**Figure 10:**
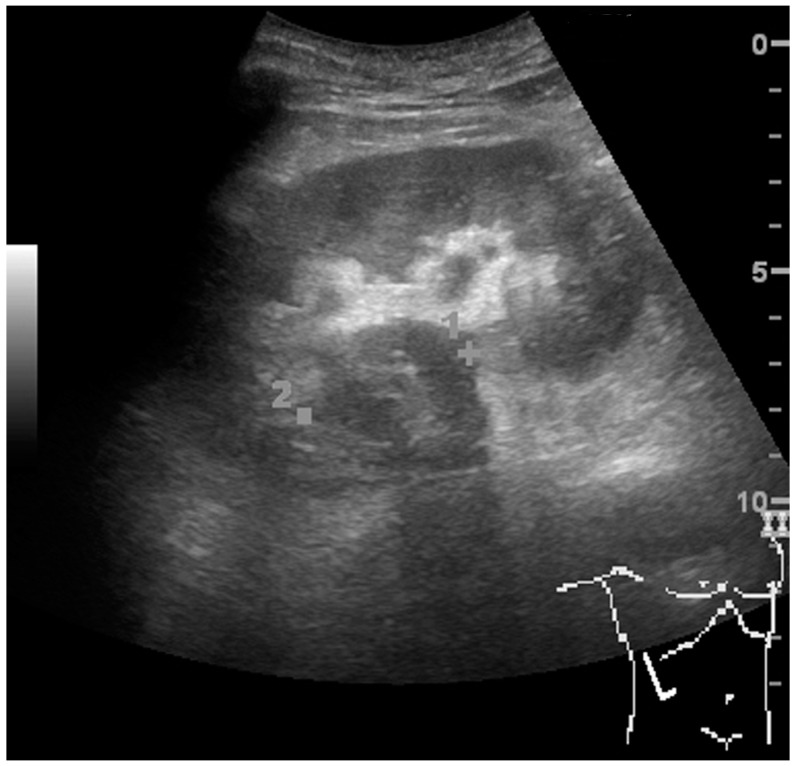
Solid tumor in the renal sinus seen as a hypoechoic mass, later found to be lymphoma. The ‘1’ and ‘2’ on the US image are reference points used for CT fusion (not shown).

Other malignant tumors in the kidney are transitional cell carcinoma and squamous cell carcinoma, which arise from the urothelium and are found the renal sinus, as well as adenocarcinoma, lymphoma and metastases, which can be found anywhere in the kidney (Figure 10) [2,4].

**Figure 11:**
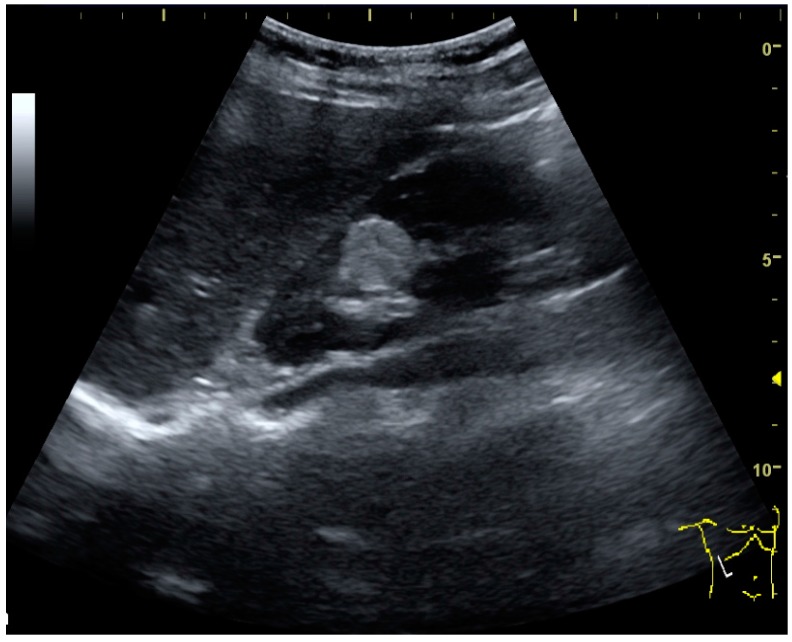
Angiomyolipoma seen as a hyperechoic mass in the upper pole of an adult kidney.

**Figure 12:**
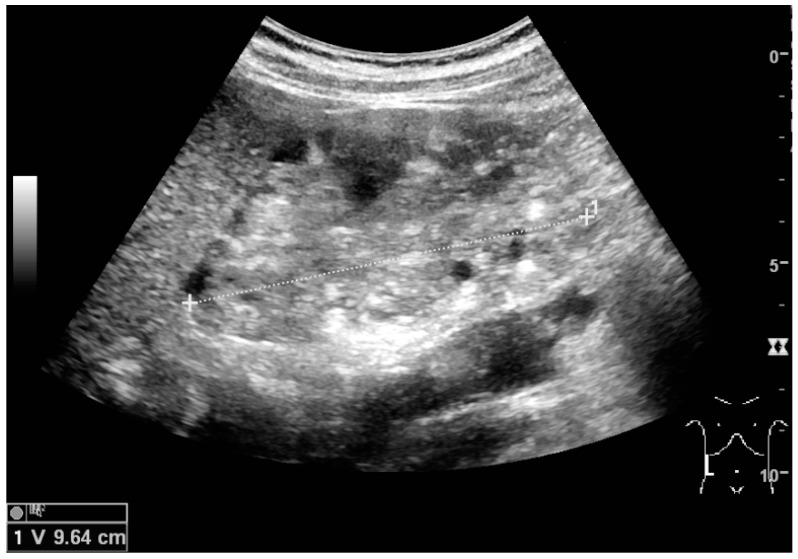
Patient with tuberous sclerosis and multiple angiomyolipomas in the kidney. Measurement of kidney length on the US image is illustrated by ‘+’ and a dashed line.

Benign solid tumors of the kidney are oncocytoma and angiomyofibroma. Oncocytoma has a varying ultrasonic appearance, but may have a central scar or calcification as a hallmark [21]. Angiomyofibroma are often found in patients with tuberous sclerosis. They are composed of fat, smooth muscle tissue and vascular elements. The echogenicity is governed by the composition of these elements, but the lesion is often hyperechoic (Figure 11 and Figure 12) [22].

Benign tumors are difficult to separate from malignant tumors using US. Thus, solid renal masses found on US are difficult to classify and should be further evaluated with CT. In special cases of cystic or solid renal masses, additional US guided biopsy or drainage is performed to identify the histologic tumor type before a decision on surgery is made [23].

## 6. Hydronephrosis

One of the primary indications for referral to US evaluation of the kidneys is evaluation of the urinary collecting system [4]. Enlargement of the urinary collecting system is usually related to urinary obstruction and can include the pelvis, the calyces and the ureter. Hydronephrosis is seen as an anechoic fluid-filled interconnected space with enhancement within the renal sinus, and normally, the dilated pelvis can be differentiated from the dilated calyces.

**Figure 13:**
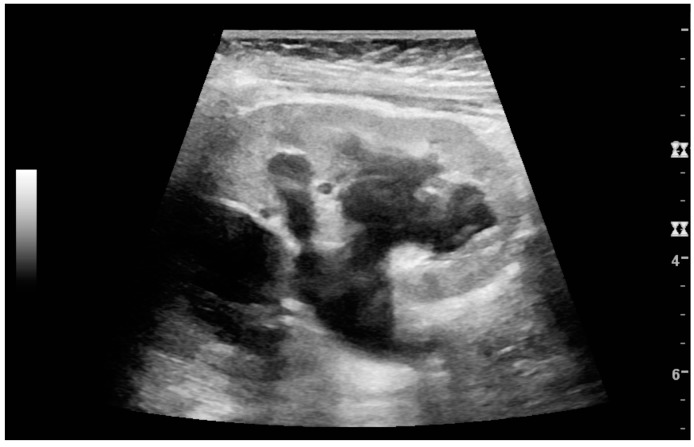
Hydronephrosis due to ureteropelvic junction obstruction in a pediatric patient.

Several conditions can result in urinary obstruction. In both adults and children, masses, such as abscesses and tumors, can compress the ureter. In children, hydronephrosis can be caused by ureteropelvic junction obstruction, ectopic inserted ureter, primary megaureter and posterior urethral valve (Figure 13). In the latter, both kidneys will be affected. In adults, hydronephrosis can be caused by urolithiasis, obstructing the outlet of the renal pelvis or the ureter, and compression of the ureter from, e.g., pregnancy and retroperitoneal fibrosis [24]. Urolithiasis is the most common cause of hydronephrosis in the adult patient and has a prevalence of 10%–15% [25].

**Figure 14:**
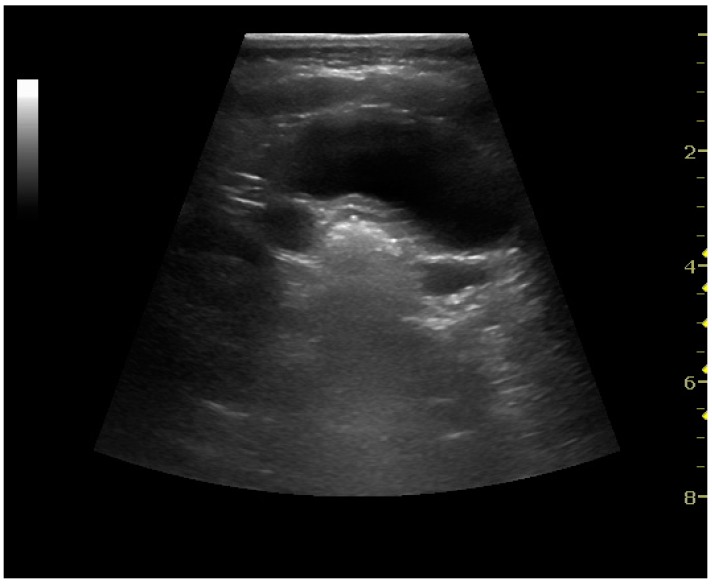
Bilateral dilatation of the ureters due to vesicoureteric reflux in a pediatric patient.

Under normal conditions, the ureter is not seen with US. However, in, e.g., urinary obstruction and vesicoureteric reflux with dilation of the ureter, the proximal part in continuation with the renal pelvis, as well as the distal part near the ostium can be evaluated (Figure 14).

**Figure 15:**
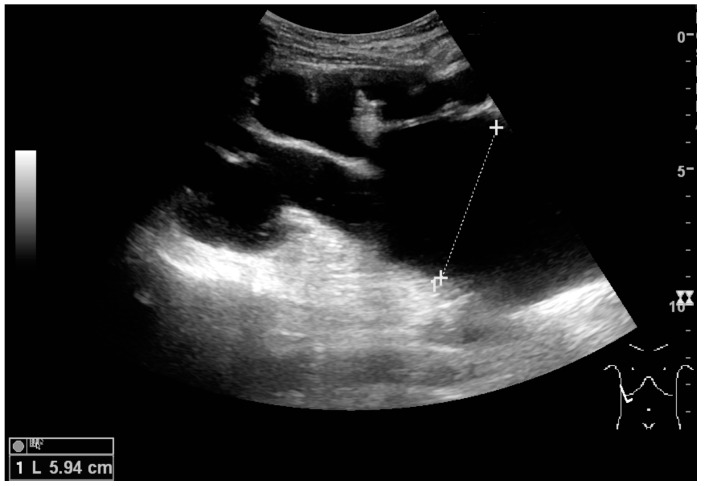
End-stage hydronephrosis with cortical thinning. Measurement of pelvic dilatation on the US image is illustrated by ‘+’ and a dashed line.

**Figure 16:**
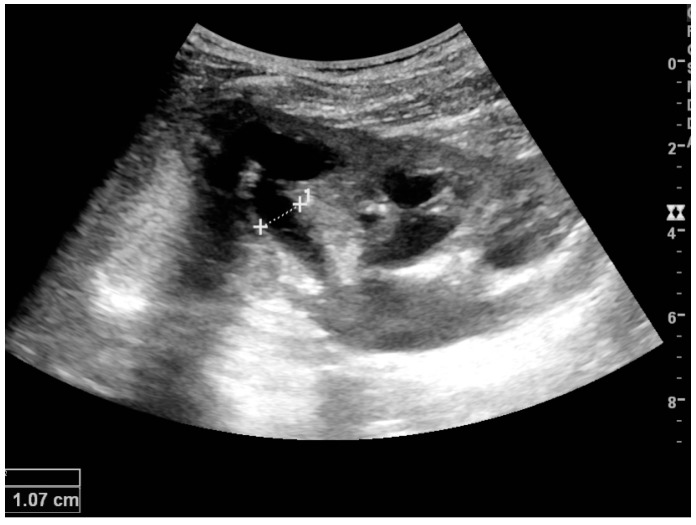
Hydronephrosis with dilated anechoic pelvis and calyces, along with cortical atrophy. The width of a calyx is measured on the US image in the longitudinal scan plane, and illustrated by ‘+’ and a dashed line.

**Figure 17:**
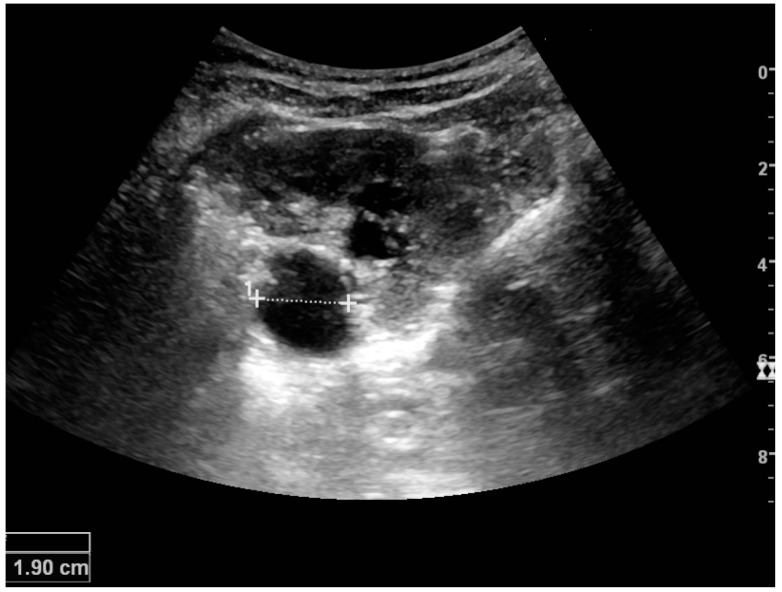
Same patient as in Figure 16 with measurement of the pelvis dilation in the transverse scan plane illustrated on the US image with ‘+’ and a dashed line.

The hydronephrosis is typically graded visually and can be divided into five categories going from a slight expansion of the renal pelvis to end-stage hydronephrosis with cortical thinning (Figure 15) [16]. The evaluation of hydronephrosis can also include measures of calyces at the level of the neck in the longitudinal scan plane, of the dilated renal pelvis in the transverse scan plane and the cortical thickness, as explained previously (Figure 16 and Figure 17) [4].

If the fluid in the dilated collecting system has echoes, pyonephrosis should be excluded by clinical exam, blood analysis and, in special cases, puncture or drainage. Hydronephrosis can also be caused by non-obstructive conditions, such as brisk diuresis in patients treated with diuretics, in pregnant women and in children with vesicoureteral reflux [24].

## 7. Renal Calculi

Even though US has a lower sensitivity and specificity than CT for the detection of urolithiasis, US, if available, is recommended as the initial imaging modality in patients with renal colic and suspected urolithiasis [26,27]. US has no risk of radiation, is reproducible and inexpensive, and the outcome is not significantly different for patients with suspected urolithiasis undergoing initial US exam compared to patients undergoing initial CT exam [26,28].

**Figure 18:**
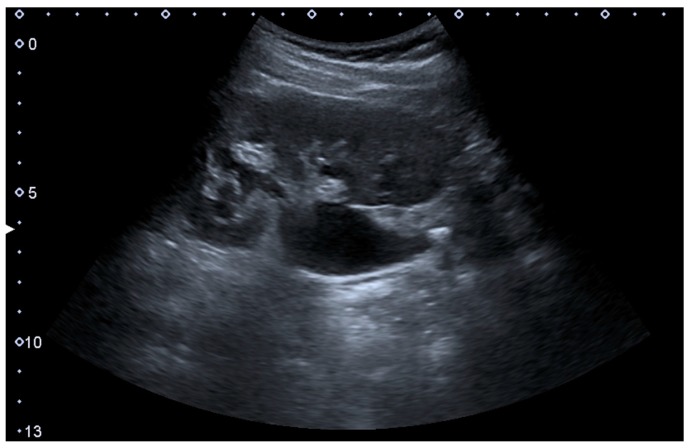
Renal stone located at the pyeloureteric junction with accompanying hydronephrosis.

**Figure 19:**
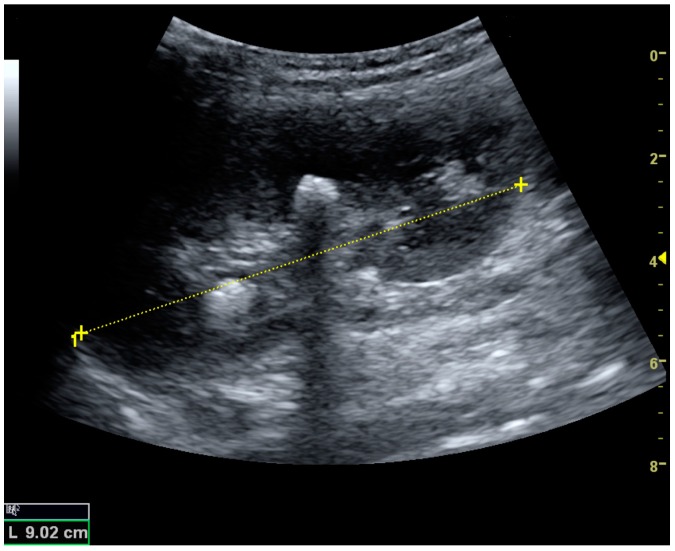
Centrally-located stone with posterior shadowing. No hydronephrosis is present. Measurement of kidney length on the US image is illustrated by ‘+’ and a dashed line.

**Figure 20:**
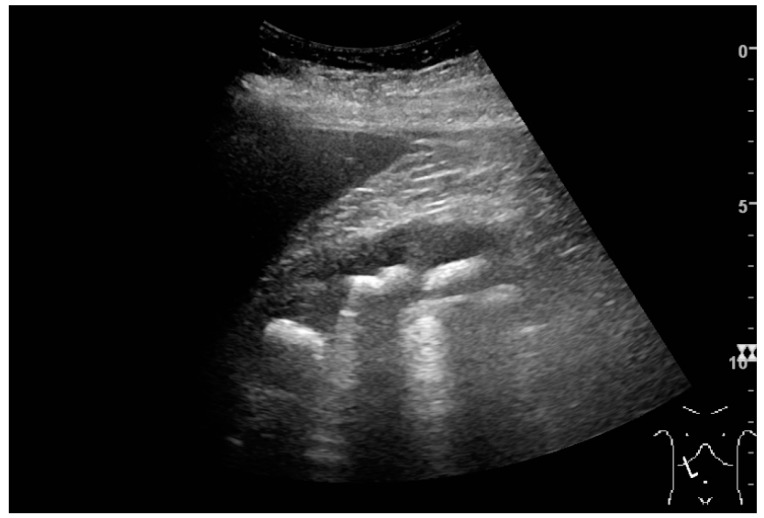
Staghorn calculi filling the entire collecting system and creating pronounced shadowing.

**Figure 21:**
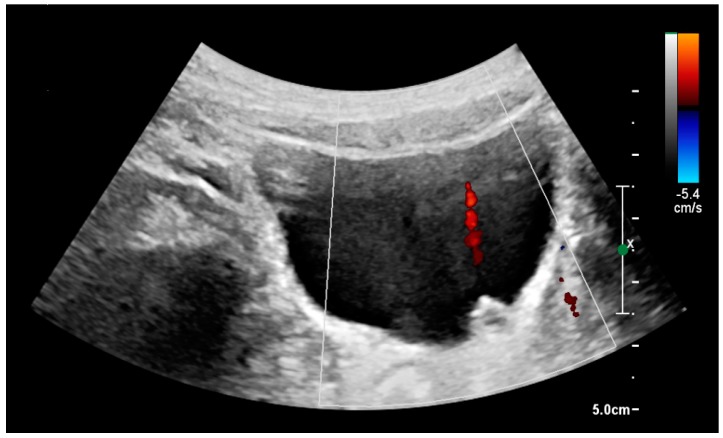
Left hydroureter with ureteric jet. No stone is visible. The red color in the color box represents motion towards the transducer as defined by the color bar.

With US, larger stones (>5–7 mm) within the kidney, *i.e.*, in the calyces, the pelvis and the pyeloureteric junction, can be differentiated, especially in the cases with accompanying hydronephrosis (Figure 18 and Figure 19) [26,29]. Hyperechoic stones are seen with accompanying posterior shadowing. Additional twinkling artifacts below the stone can often be seen using Doppler US [30]. Large stones filling the entire collecting system are called coral stones or staghorn calculi and are easily visualized with US (Figure 20). Stones in the ureters are usually not visualized with US due to the air-filled intestines obscuring the insonation window. However, ureteral stones near the ostium can be visualized with a scan position over the bladder. An exam of the ureteric orifices and the excretion of urine to the bladder can be performed by inspecting the ureteric jets in the bladder with color Doppler US (Figure 21) [4,24].

## 8. Chronic Kidney Disease

**Figure 22:**
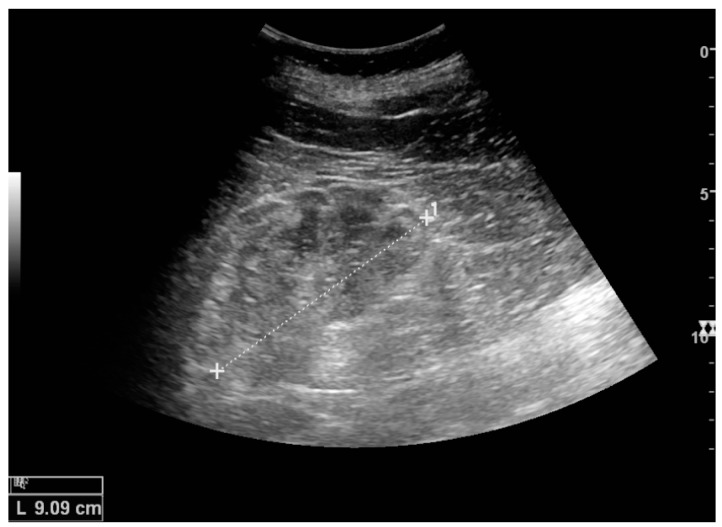
Chronic renal disease caused by glomerulonephritis with increased echogenicity and reduced cortical thickness. Measurement of kidney length on the US image is illustrated by ‘+’ and a dashed line.

**Figure 23:**
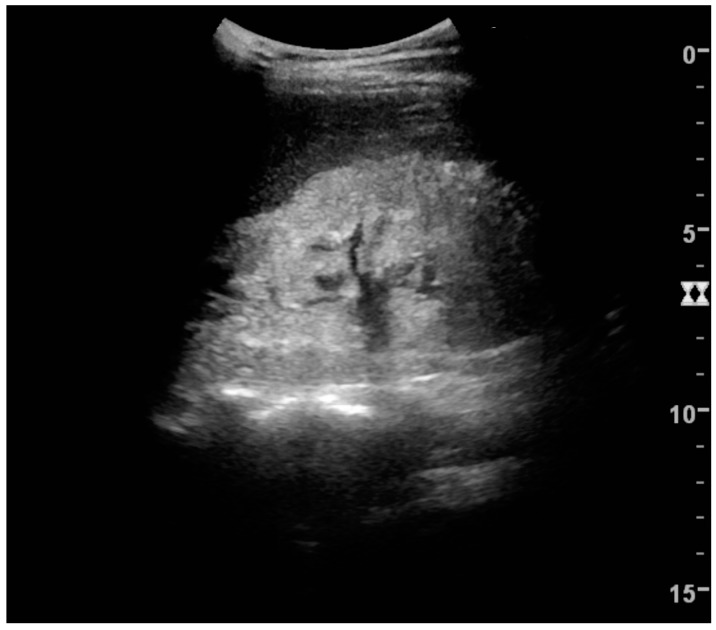
Nephrotic syndrome. Hyperechoic kidney without demarcation of cortex and medulla.

**Figure 24:**
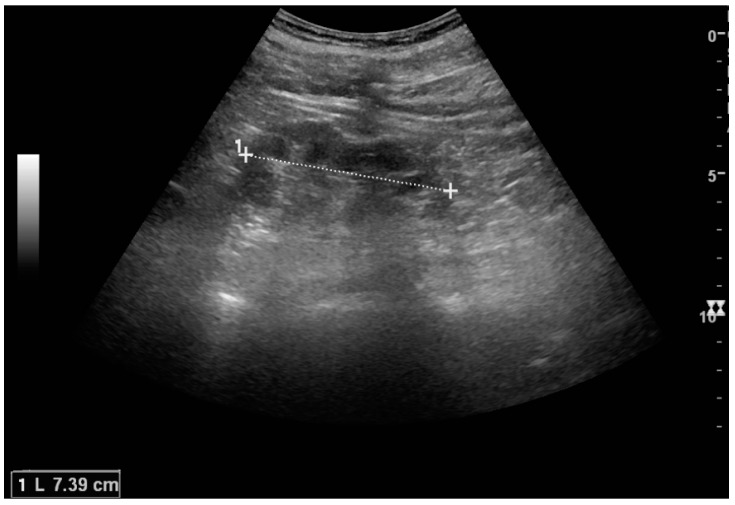
Chronic pyelonephritis with reduced kidney size and focal cortical thinning. Measurement of kidney length on the US image is illustrated by ‘+’ and a dashed line.

**Figure 25:**
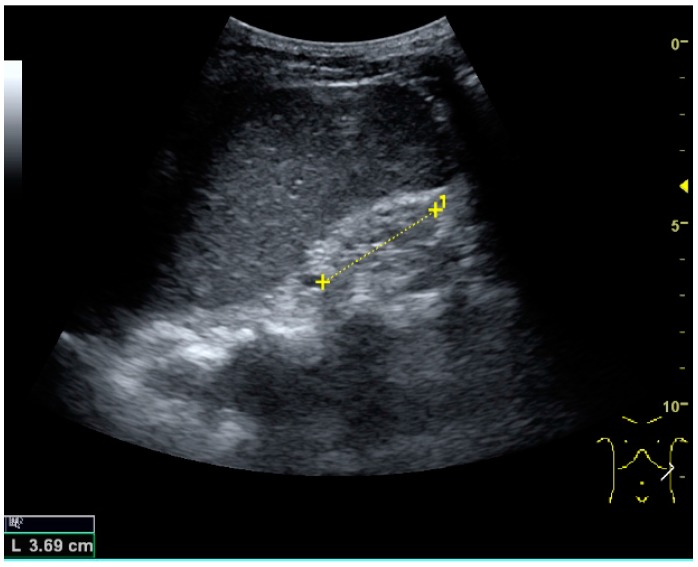
End-stage chronic kidney disease with increased echogenicity, homogenous architecture without visible differentiation between parenchyma and renal sinus and reduced kidney size. Measurement of kidney length on the US image is illustrated by ‘+’ and a dashed line.

US is useful for diagnostic and prognostic purposes in chronic kidney disease. Whether the underlying pathologic change is glomerular sclerosis, tubular atrophy, interstitial fibrosis or inflammation, the result is often increased echogenicity of the cortex. The echogenicity of the kidney should be related to the echogenicity of either the liver or the spleen (Figure 22 and Figure 23) [2]. Moreover, decreased renal size and cortical thinning are also often seen and especially when disease progresses (Figure 24 and Figure 25). However, kidney size correlates to height, and short persons tend to have small kidneys; thus, kidney size as the only parameter is not reliable [2].

## 9. Acute Renal Injury

**Figure 26:**
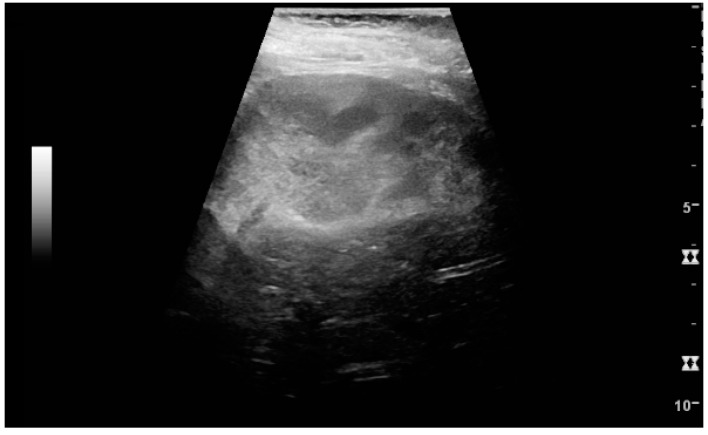
Acute pyelonephritis with increased cortical echogenicity and blurred delineation of the upper pole.

**Figure 27:**
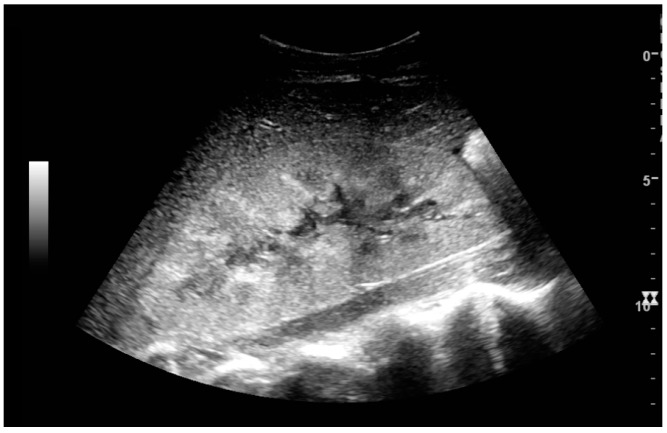
Postoperative renal failure with increased cortical echogenicity and kidney size. Biopsy showed acute tubular necrosis.

**Figure 28:**
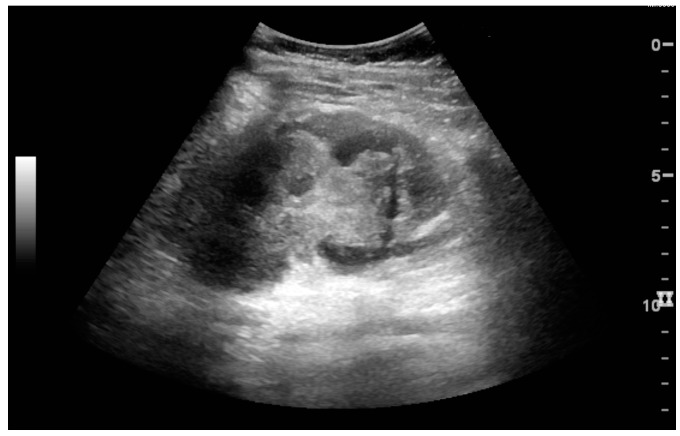
Renal trauma with laceration of the lower pole and subcapsular fluid collection below the kidney.

The acute changes in the kidney are often examined with US as the first-line modality, where CT and magnetic resonance imaging (MRI) are used for the follow-up examinations and when US fails to demonstrate abnormalities [31]. In evaluation of the acute changes in the kidney, the echogenicity of the renal structures, the delineation of the kidney, the renal vascularity, kidney size and focal abnormalities are observed (Figure 26 and Figure 27). CT is preferred in renal traumas, but US is used for follow-up, especially in the patients suspected for the formation of urinomas [32] (Figure 28).

## 10. US-Guided Intervention

Sonography is the modality of choice for guidance when performing intervention in the kidney, whether it is kidney biopsy, percutaneous nephrostomy or abscess drainage. Historically, thermal ablation of renal tumors is performed under CT guidance, as the risk of injuring neighboring intestines during the US-guided procedure was considered too high due to poor identification of the moving bowels [4,23]. However, recent guidelines for renal interventional US include radiofrequency, microwave and cryoablation with US as the ideal imaging guide [33,34].

**Figure 29:**
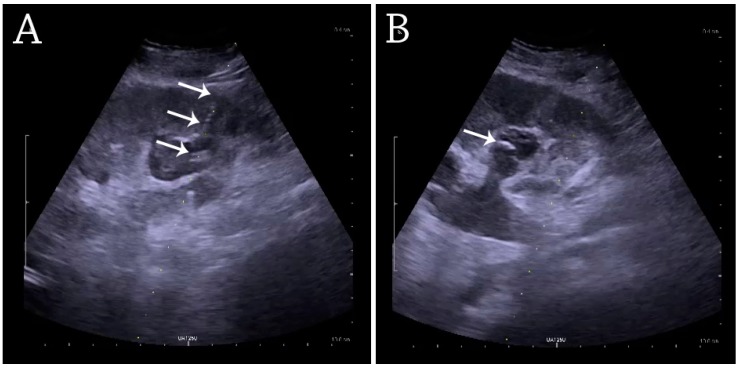
(**A**) Percutaneous nephrostomy tube placed through a calyx in the lower pole of a kidney with hydronephrosis. (**B**) The pigtail catheter is placed in the dilated calyx. The tube in (A) and the pigtail in (B) are marked with white arrows.

For percutaneous nephrostomy and abscess drainage, either the one-step or the Seldinger technique is used. Using the Seldinger technique, the cavity is punctured with a sharp hollow needle, called a trocar. A round-tipped guidewire is then advanced through the lumen of the trocar, and after withdrawal of the trocar, a catheter or nephrostomy can be inserted over the guidewire to ensure correct placement. The one-step technique is when insertion of the drain or nephrostomy is done without the aid of a guidewire [4]. The interventions are performed under local anesthesia and in a sterile setup. The procedures can be carried out with or without needle guidance according to preference, experience and setup (Figure 29) [33,34].

## 11. CEUS, Image Fusion and Elastography

**Figure 30:**
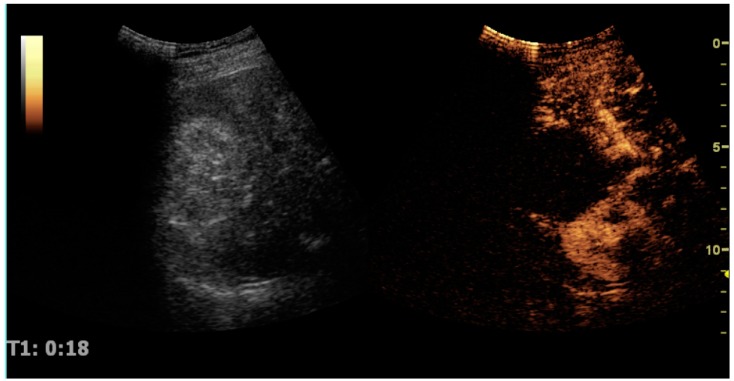
Renal cell carcinoma successfully treated with thermal ablation, as no contrast enhancement is seen.

CEUS can evaluate microvasculature, which color Doppler US is unable to detect. In renal US examination, CEUS can be used to differentiate tumor and pseudotumor, such as prominent columns of Bertin. Pseudotumors enhance as adjacent renal tissue. The use of CEUS is recommended in special cases to distinguish between cystic and hypovascularized solid lesions, to characterize complex cysts, abscesses, traumatic lesions and ischemic lesions [35,36]. Solid malignant tumors in the kidney do not exhibit specific enhancement patterns like some liver lesions, and no valid enhancement criteria between benign and malignant renal lesions have been proposed [37,38]. However, CEUS is used in some patients after ablation of renal cell carcinoma to evaluate contrast uptake in the treated area (Figure 30).

**Figure 31:**
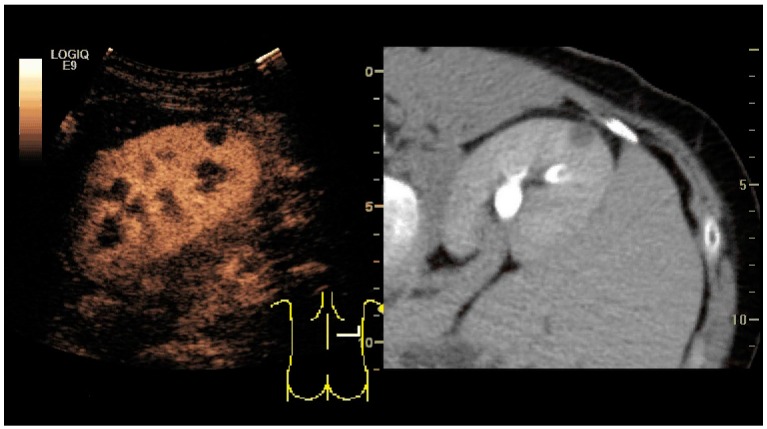
Unspecific cortical lesion on CT is confirmed cystic and benign with contrast-enhanced ultrasound (CEUS) using image fusion.

Image fusion of ultrasound with a previously recorded dataset of CT or other modalities is rarely used in renal US. Reports on image fusion using CEUS or US combined with CT or MRI in the examination of renal lesions and in difficult US-guided renal interventions have been published (Figure 31) [39]. However, no recommendations have been published so far.

**Figure 32:**
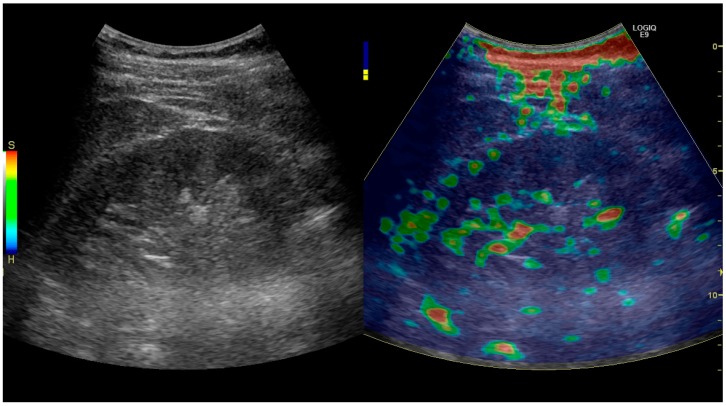
Strain elastography of a normal kidney. Red depicts soft areas, and blue depicts hard areas relative to the entire elastography image. Note that the medulla is softer than the cortex. A color bar is shown to the left of the image, where ‘S’ and ‘H’ denote soft and hard tissue, respectively.

Elastography is a US method to visualize the elasticity of tissue. Preliminary reports on US elastography used on transplanted kidneys to evaluate cortical fibrosis have been published showing promising results (Figure 32) [40].

## 12. Conclusions

Renal US is a versatile and useful examination. US is an accessible, inexpensive and fast aid for decision-making in patients with renal symptoms and for guidance in renal intervention. However, renal US has certain limitations, and other modalities, such as CT and MRI, should always be considered as supplementary imaging modalities in the assessment of renal disease.
